# Mechanical Dentistry Items

**Published:** 1880-05

**Authors:** 


### EDITORIAL, ETC.

*Mechanical Dentistry Items.*—From the transactions of the 1879 meeting of the American Dental Association, lately published, we copy the following items which the discussion on this subject brought forth.

Dr. Stockton, speaking of the advantage of gold plates, remarked :

"I think there is nothing so good for partial plates as gold. For whole plates for the lower jaw continuous gum is the best, while gold for upper sets, fastened with rubber or celluloid, is very desirable. I mention this last because you can thus have a cleaner denture than you can where the teeth are soldered on ; although the strongest and best possible work that can be made, in my judgment, is a good gold plate with single teeth soldered on. You can pack rubber in the interstices, and make it clean in that way. When rubber is done away with dentists will be better qualified to practice dentistry as it was practiced years ago. The proper instructions to students are not now given by preceptors, and I think not in colleges, judging from the fact that I see no sets of teeth that are ground up and fitted to the plate as they should be. My practice generally is to take the impression immediately after extraction, and put in a set on celluloid, allowing them to be worn as long as they can be comfortably, and then, if necessary, before the alveolus is absorbed sufficiently for the permanent set, to make them over again. When the time comes I tell my patients that it will be cheaper for them, in health, in comfort and in the pocket, to have a good set of teeth made on gold than on anything else."

Dr. Atkinson alluded to the casting of gold plates as follows :

"There is some experimenting going on with the hope of casting gold plates so as to be perfectly air and moisture tight to the teeth for artificial dentures, something after the manner of the well known cheoplasty of A. A. Blandy of years gone by.

My own impression is that they will, for partial purposes, succeed very well, and I do not know but for entire sets; but I am not so anxious to teach people how to redeem their fellow-men from mischiefs already incurred as to teach them how to avoid the necessity of redemption, to live according to the laws of hygiene, of morality, of fraternity, kindly and honestly, that we may do good by rule, and mischief only by chance and by exception, rather than going on as it is now in artificial dentistry, doing mischief by rule and doing good by chance."

Dr. Essig, alluding to the experiments of the late Dr. James B. Bean, remarked:

"Twelve years ago Dr. Bean, of Baltimore, who was then experimenting with his aluminium plates, told me it would be quite as easy to cast a gold or silver plate as one of aluminium. The specific gravity of gold or silver being so much greater than aluminium, the probabilities were that there would be less difficulty in the metal finding its way into every part of the matrix than was the case with the lighter metal aluminium. Dr. Bean's process was a very beautiful one, and it was very successful as far as the method of making went, but it was one that very few men could accomplish on account of the difficulty of the manipulation and the care that was required. It would be very much easier to swage a plate, and any skillful man who knows anything about that branch of his profession can swage a plate to fit just as perfectly as one made by casting. I see my friend Atkinson shaking his head negatively, but there is one matter that he seems to forget: a plate that is cast must be the size of the plaster model. The plaster model is always larger than the mouth. Every one knows that a plate that is made to fit exactly upon the plaster model must be a little larger than the mouth, and that is an element of trouble at once. A plate should be somewhat smaller than the mouth to compensate for the depression of the capillaries and the change and absorption that takes place along the ridge. By way of illustration, if a plate be made to fit the cast perfectly, and be then introduced into the mouth, it will, in the course of a few days, be found to press a little hard upon the roof of the mouth, and not so hard upon the ridge. That is wrong; it should press in the first place a little unduly along the ridge, and should be a little loose at the palatine arch, for the certainty is that it will in a few days adapt itself to the ridge, while the palatine arch is stable.

That is the result of the experience of everybody, I think, who has watched the matter closely."

Dr. Rehwinkel alluded to a method of overcoming the heating which celluloid and rubber plates occasion in the mouth, by lining the plates with a thin plate of gold. He described the method as follows:

"On the outside there is an attachment with a very narrow strip of platinum twisted up in the shape of a spiral spring and brought out into a coil. It is simply put around the edge, so that the rubber will embrace it and become thoroughly and firmly attached all around the edge. In vulcanite or celluloid the plate is put over the model and pressed into the matrix. You will find that this device will do away with the tendency to sore mouth and remedy the difficulty where a trouble of that kind does exist; at least it has succeeded with me in three very marked cases."

